# Fetal and neonatal bilirubin metabolism

**DOI:** 10.3389/fped.2022.1002408

**Published:** 2023-02-07

**Authors:** Susumu Itoh, Hitoshi Okada, Kosuke Koyano, Shinji Nakamura, Yukihiko Konishi, Takashi Iwase, Takashi Kusaka

**Affiliations:** ^1^ Department of Pediatrics, Faculty of Medicine, Kagawa University, Kagawa, Japan; ^2^ Division of Analytical Technology, Department of Medical Technology, Kagawa Prefectural University of Health Sciences, Kagawa, Japan; ^3^ Maternal Perinatal Center, Faculty of Medicine, Kagawa University, Kagawa, Japan

**Keywords:** bilirubin photoisomers, breast milk jaundice, human serum albumin, neonatal jaundice, physiological effects, reactive oxygen species, *4Z,15Z*-bilirubin IXα, oxidation products

## Abstract

Human fetal and neonatal bilirubin metabolism is centered on *4Z,15Z*-bilirubin IXα (BR) due to the extremely low BR conjugating capacity of the liver. BR is a unique, highly lipophilic substance with physiological and toxic effects in the cell membranes of organs and body tissues. The fetus excretes BR through the placenta to the maternal circulation. After birth, BR is thought to act as an antioxidant against the increase in reactive oxygen species caused by the rapid increase in oxygen concentration during the adaptation process from in amniotic fluid to in air. However, bilirubin encephalopathy is a toxic effect of bilirubin. Due to the lipophilic nature of BR, it must be bound to a carrier to be distributed to various parts of the body by hydrophilic blood. This carrier of BR is human serum albumin (HSA). In humans, BR can be excreted efficiently after undergoing photochemical reactions upon high affinity binding to HSA. HSA also plays an important role in the prevention of bilirubin encephalopathy. This review focuses on the developmental and physiological role of bilirubin metabolism during the fetal and neonatal periods.

## 1. Introduction

*4Z,15Z*-Bilirubin IXα (BR) and human serum albumin (HSA) have unique chemical structures that underlie their physiological importance in humans. Nonetheless, their *in vivo* actions are not fully understood. In particular, bilirubin has been the focus of considerable attention because it causes bilirubin encephalopathy. In 1954, Bernhard et al. reported that bilirubin stabilizes and improves the absorption of vitamin A and linoleic acid, a polyunsaturated fatty acid, in the intestinal tract (1). The stabilizing effect of bilirubin on oxidizable substances was subsequently reported (2–5). The antioxidant properties of bilirubin have also gained attention in terms of its physiological effects in preterm and term infants who have weak protection against activated oxygen as well as in terms of why humans in particular develop neonatal jaundice (6, 7). BR binds with high affinity to HSA, which is responsible for the very low levels of free BR in the blood and its wide distribution in the body (8, 9). However, HSA is uniquely evolved compared with the serum albumin in non-human primates in terms of its photochemical reaction with BR (10–13). This review aims to elucidate the following basic issues, with a focus on physiological effects: (1) the chemical structure and metabolism of BR; (2) the oxidation products of BR; (3) the photochemical reaction of BR in phototherapy for neonatal hyperbilirubinemia; (4) fetal bilirubin metabolism; and (5) neonatal bilirubin metabolism.

## 2. Chemical structure and metabolism of *4Z,15Z*-bilirubin IXα

BR is a tetrapyrrole with four imino groups, two carbonyl groups, and two propionic acid groups. Despite the presence of the many polar groups, BR is extremely insoluble in water. This is due to the intramolecular hydrogen bonding of these polar groups and the presence of hydrophobic groups such as methyl and vinyl groups on the surface of the BR molecule (14, 15). The cleavage of protoheme IX (heme B) at the α position is essential for the formation of the BR structure, and the substrate specificity of the cleavage enzyme, heme oxygenase, is vital. This specificity is reported to be due to the electrostatic interaction of the propionate groups of heme B with Lys-18, Lys-179, and Arg-183 of the heme oxygenase protein and hydrogen bonding with Tyr-134, which fixes the α–γ axis of heme B, resulting in a fixed binding direction between heme B and heme oxygenase (16, 17).

For BR to be distributed throughout the body, it needs a carrier in the hydrophilic bloodstream. Its hydrophobic physicochemical properties make it suitable for distribution in lipids, mainly in cell membranes. HSA serves this purpose. BR is thought to bind with high affinity to HSA at domain IIA with a binding constant on the order of 10^7^ and to be carried and distributed to various organs and tissues where it exerts antioxidant effects (18–20). The amino acid pocket of HSA is a salt-type right-hand chirality enantiomer with some of the BR hydrogen bonds broken and hydroxyl groups displayed, and it is ionically bound with high affinity to Lys-190 and hydrophobic bonding to hydrophobic amino acids (21, 22). The binding of BR to HSA allows for unique photochemical reactions due to the asymmetric structure of the dipyrrole facing its methylene bridge and the conformation of the bound BR (see Photochemistry of *4Z,15Z*-bilirubin IXα in phototherapy for neonatal hyperbilirubinemia).

The antioxidant effect of BR involves both scavenging and quenching activity. In the former pathway, BR combines with reactive oxygen species (ROS) to form oxidation products, and in the latter pathway, BR is oxidized by electron transfer to produce biliverdin, which can be metabolized back to BR by biliverdin reductase, a widely distributed enzyme in the body. However, it is difficult to assess the *in vivo* antioxidant activity of this cycle (23).

## 3. Oxidation products of *4Z,15Z*-bilirubin IXα

**Figure 1:**
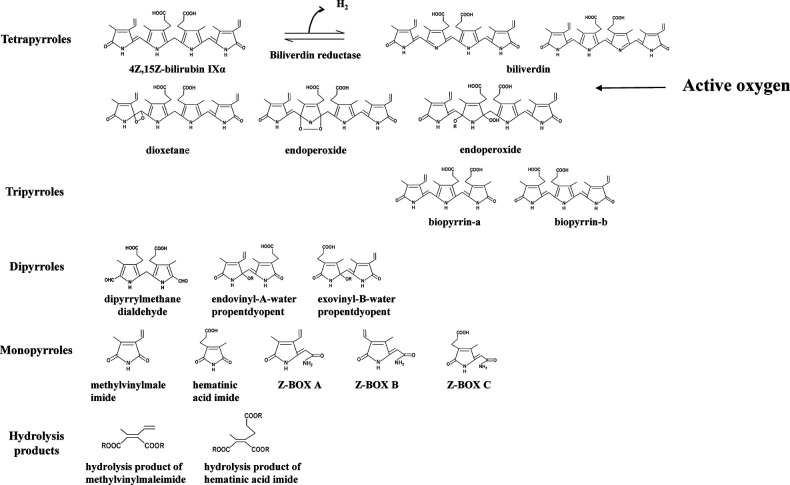
*4Z,15Z*-bilirubin IXα and bilirubin oxidation products. The bilirubin oxidation products formed from *4Z,15Z*-bilirubin IXα are divided into tetrapyrroles, tripyrroles, dipyrroles, monopyrroles, and hydrolysis products with their corresponding structures. Bilirubin oxidation products produced by quenching activity: biliverdin. Bilirubin oxidation products produced by scavenging activity: oxidizing substances other than biliverdin. The abbreviations and the articles in which they are listed: biliverdin (23–26); biopyrrin-a and biopyrrin-b (27); exovinyl-B-water propentdyopent, endovinyl-B-water propentdyopent, and hematinic acid imide (28); Z-BOX, Z-bilirubin oxidant product; Z-BOX A and Z-BOX B (29); Z-BOX C (30); hydrolysis product of methylvinylmaleimide and hematinic acid imide (28); and exovinyl-B-water propentdyopent & endovinyl-B-water propentdyopent [propentdyopents determined by pentdyopent reaction (23, 31)].

To validate the evidence indicating that BR acts as an antioxidant, it is necessary to demonstrate both a decrease in BR and an increase in BR oxidation products resulting from the scavenging and quenching of ROS. Therefore, the identification of BR and its oxidation products is important. The oxidation products of BR are shown in Figure 1 and comprise mainly tetrapyrroles, tripyrroles, dipyrroles, and monopyrroles and their oxidized metabolites (27–30). In the past, it was thought that BR was itself a photosensitizer and that this oxidative pathway was the primary route of BR metabolism in phototherapy for neonatal hyperbilirubinemia (32). However, it was clarified that the photochemical reactions of BR are not type II photochemical reaction in which energy is transferred from the photoexcited state of BR to triplet oxygen to generate singlet oxygen that oxidizes BR (33, 34). Elucidation of the photochemical reactions of BR revealed that the main reactions are geometric isomerization and subsequent structural isomerization, while the aforementioned tetrapyrrole oxidation products remain intact (see Photochemistry of *4Z,15Z*-bilirubin IXα in phototherapy for neonatal hyperbilirubinemia).

Turning to the BR oxidation products, biliverdin produced by quenching activity (electron transfer only) and the bilirubin oxidation products produced by scavenging activity (reaction with ROS) have been detected *in vivo*. The following products have been identified: biliverdin (24–26), biotripyrrin-a (27), biotripyrrin-b (27), exovinyl-B-water propentdyopent (28), endovinyl-B-water propentdyopent (28), hematinic acid imide (28), Z-BOX A (29), Z-BOX B (29), Z-BOX C (30), and a hydrolysis product of methylvinylmaleimide and hematic acid imide (28).

## 4. Photochemistry of *4Z,15Z*-bilirubin IXα in phototherapy for neonatal hyperbilirubinemia

**Figure 2:**
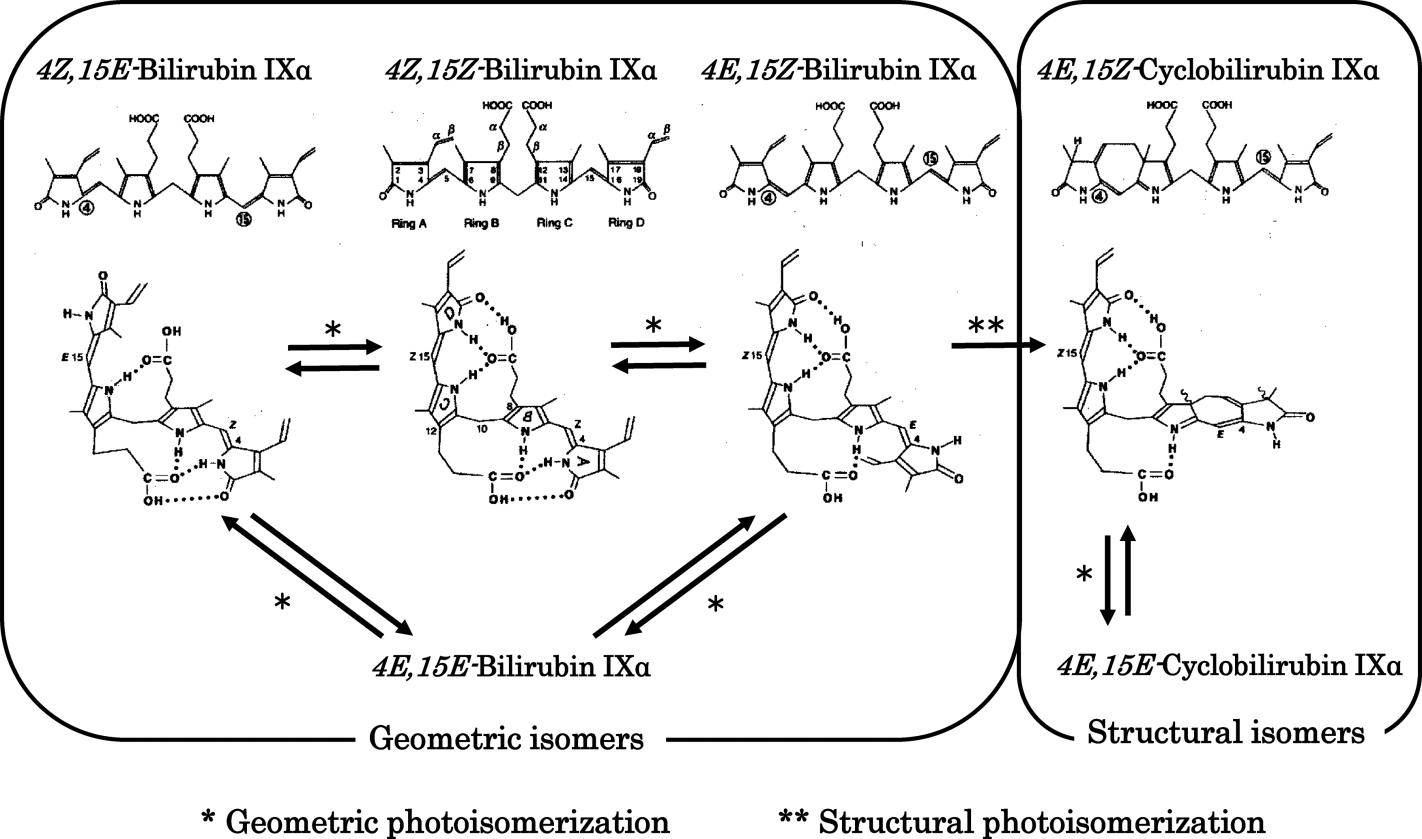
Photochemical changes in *4Z,15Z*-bilirubin IXα. Photochemical changes in *4Z,15Z*-bilirubin IXα in bilirubin–human serum albumin are predominantly to *4Z,15E*-bilirubin IXα and cyclobilirubin IXα (lumirubin). Storage route of bilirubin: *4Z,15E*-Bilirubin IXα is excreted from the liver into the bile and then may reconvert to *4Z,15Z*-bilirubin IXα in the digestive tract to be retained in the body through the enterohepatic circulation. Elimination route of bilirubin: *4E,15Z*-Cyclobilirubin IXα (Z-lumirubin) and *4E,15E*-cyclobilirubin IXα (E-lumirubin) are the most important pathways affecting the clinical efficacy of phototherapy because they polymerize into a dark brown substance that causes bronze baby syndrome, an adverse reaction to phototherapy, and is ultimately excreted from the body.

The major photochemical reaction of BR occurring during phototherapy is shown in Figure 2 (35–38). BR bound with high affinity to domain IIA of HSA undergoes specific photochemical reactions, which is explained in the section on physiological aspects in this review. The major product of the geometric isomerization reaction is *4Z,15E*-bilirubin IXα, while the major product of the structural isomerization reaction is *4E,15Z*-cyclobilirubin IXα (*Z*-lumirubin) (39). *4Z,15E*-Bilirubin IXα moves to domain IB, where HSA binding is weak (40), and the levels of its free form increase. This form is excreted from the liver into the bile and then may reconvert to BR in the digestive tract to be retained in the body through the enterohepatic circulation. BR excreted into the intestinal tract is thought to play a role in antioxidant activity. In blood, the amount of BR bound to domain IIA of HSA is reduced and toxic free BR can bind again at the domain II site, resulting in a decrease in free BR (10, 41–43).

*4E,15Z*-Cyclobilirubin IXα is the most important compound formed during phototherapy because it polymerizes into a dark brown substance that causes bronze baby syndrome, an adverse reaction to phototherapy, and is ultimately excreted from the body (44, 45). In particular, the rate of formation of *4E,15Z*-cyclobilirubin IXα *via 4E,15Z*-bilirubin IXα from BR bound to HSA is much higher than for other albumins in non-human primates (10–13, 46–50). Phototherapy LED light centered at turquoise and green wavelengths produces more cyclobilirubin and less *4Z,15E*-bilirubin (50, 51). The asymmetric structure of the dipyrroles centered on the methylene bridge of BR is important for effectively achieving both geometric and structural isomerization reactions. In contrast, it is difficult to achieve these reactions for the symmetric *4Z,15Z*-bilirubin IIIα and XIIIα (52). In solution with various acid catalysts, BR can undergo rearrangement to a mixture bilirubin IIIα, BR and XIIIα through structural isomerization (intermolecular dipyyrole exchange) (53). This isomerization reaction has caused many erroneous results *in vitro* (54), but plays no important role in bilirubin photoisomerization *in vivo* (55).

In the bilirubin oxidation pathway, the total excretion of BR oxidation products during phototherapy is very low (28). Before phototherapy, hematinic acid imide and hydrolysis products of methylvinylmaleimide and hematinic acid imide are detected by high-performance liquid chromatography. They increase during phototherapy, and exovinyl-B-water propentdyopent and endovinyl-B-water propentdyopent are present as well (28). We speculate that BR is oxidized and acts as an antioxidant even before phototherapy. According to a previous report (28), the increase in these BR oxidation products during phototherapy is considered to reflect the BR antioxidant activity. This increase indirectly indicates an increase in ROS generated endogenously by photosensitizers such as riboflavin, which is highly photosensitizing in the blue wavelength region (56, 57). Irradiation of a BR–HSA complex solution containing riboflavin with blue-white light induces a greater increase in BR oxidation products compared with green fluorescent light (58). The wavelength characteristics of the phototherapy light source are important for clinical efficacy and green light also has sufficient clinical benefits (51, 59–62). However, the photosensitizing effect of endogenous substances as an adverse reaction is also important, and green light is thought to lessen the severity of this adverse reaction compared with conventional blue light (58).

## 5. Fetal bilirubin metabolism

During the fetal period, bilirubin-producing enzymes and bilirubin-conjugating enzymes are developmentally specific. The substrate specificity of the heme oxygenase that produces biliverdin is important (63, 64). From 14 to 15 weeks of gestation, bilirubin IXβ, which cannot form intramolecular hydrogen bonds, is the major component; it is excreted in bile and urine in an intact form without conjugation (65, 66). Bilirubin IXα is present at 16–17 weeks of gestation, but bilirubin IXβ predominates until 20 weeks of gestation (65). Bilirubin IXδ, bilirubin IXγ, and biliverdin also have physicochemical properties similar to those of bilirubin IXβ (66, 67), but the physiological significance of bilirubin IXβ during development is unknown.

**Figure 3:**
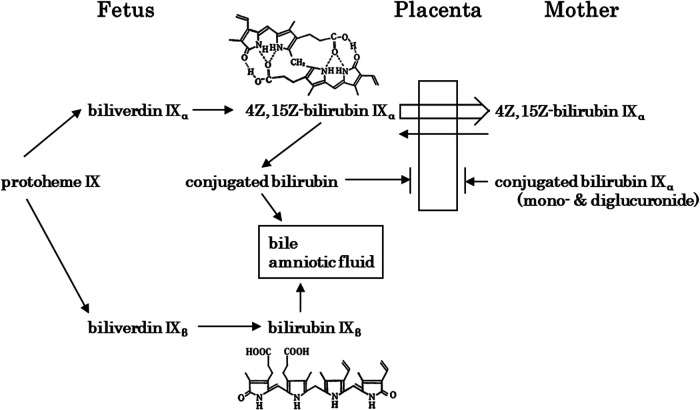
Fetal bilirubin metabolism [modified in part from itoh (65)]. Bilirubin IXβ, which is produced in early fetal life, is hydrophilic and is excreted *via* the gastrointestinal tract and amniotic fluid. In contrast, *4Z,15Z*-bilirubin IXα, which is produced later in life, is hydrophobic and crosses the placenta for excretion by the mother. At the fetal stage, the extremely low activity of *4Z,15Z*-bilirubin IXα-conjugating enzymes is important.

The development of BR conjugation leads to the presence of xylose, glucose, and unidentified monoconjugated bilirubin at around 20 weeks of gestation, with monoglucuronosyl bilirubin appearing only at 20–23 weeks in bile. Bilirubin monoconjugated to glucose or xylose predominates up to 30 weeks, while monoglucuronosyl bilirubin becomes the predominant form at term (65). Bilirubin UDP-glucuronosyltransferase activity in the fetal human liver is 1/1,000 of that in adults until 30 weeks of gestation, after which it increases to 1/100 of that at term (68). Thus, hepatic bilirubin UDP-glucuronosyltransferase activity is extremely low during the fetal period. An overview of fetal bilirubin metabolism is shown in Figure 3 (69). Bilirubin IXβ and conjugated bilirubin are substances that accumulate in the fetal intestine and amniotic fluid, whereas bilirubin IXα is excreted by the mother after crossing the placenta. In this regard, extremely low hepatic bilirubin UDP-glucuronosyltransferase activity and placental function are important for the physiology of fetal developmental. In contrast, the physiological effects of the bilirubin IXβ that accumulates in the fetal intestine and amniotic fluid have not been elucidated, although a possibility is that it has a stabilizing effect on easily oxidized substances (1–4). Abnormalities in fetal bilirubin metabolism include fetal hemolytic disease due to Rh-incompatible hemolytic disease on the fetal side. If placental function is normal, fetal hyperbilirubinemia is not a concern because the fetus excretes BR through the placenta to the maternal circulation, although fetal hydrops due to severe anemia is a problem (70). Maternal Dubin-Johnson syndrome has a high rate of miscarriage (71). In Gunn rats, maternal hyperbilirubinemia has been reported to decrease pregnancy rates (72). In humans, maternal hyperbilirubinemia increases preterm birth rates (73), but the prognosis for the infants is better in late pregnancy (74).

## 6. Bilirubin metabolism in neonates

**Figure 4:**
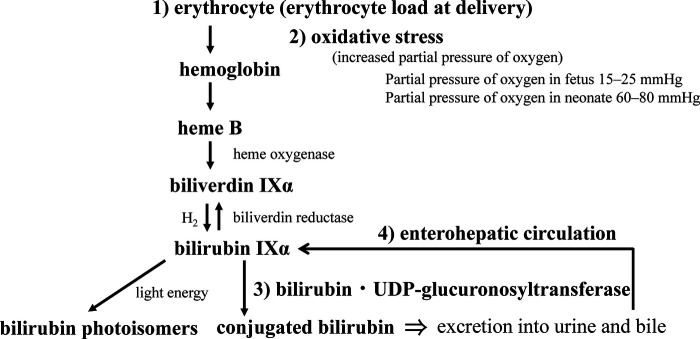
Neonatal bilirubin metabolism. The perinatal metabolism of bilirubin is summarized in terms of (1) bilirubin load, (2) its response to oxidative stress at birth, (3) the development of conjugating enzymes, and (4) the significance of the enterohepatic circulation (see text for details).

After birth, maternal excretion of bilirubin from the placenta no longer occurs, and the neonate’s own ability to metabolize and excrete bilirubin is required. In that process, physiological neonatal jaundice can develop, which involves the production, excretion, and enterohepatic circulation of BR. Bilirubin production in neonates is reported to be 8.5 mg/kg/day, about twice that in adults (75). This is due to the shorter erythrocyte lifespan of about 52 days in neonates (76) versus about 120 days in adults (77, 78). Placental transfusion of red blood cells to infants occurs at birth, which acts as a source of BR. Approximately 20 ml/kg of blood flows to the infant from the placenta by the time the umbilical cord pulsation stops (79). The BR load is calculated by assuming that the baby weighs 3 kg and that the hemoglobin (Hb) concentration of the cord blood is 15 g/dL. The following is a summary of the formula (Figure 4).

$$\begin{array}{rl} \mathrm{Bilirubin}\;\mathrm{load} & =15\;g/\mathrm{dL}\;(\mathrm{Hb}\;\mathrm{concentration})\\ & \;\times \{585\;(\mathrm{molecular}\;\mathrm{weight}\;\mathrm{of}\;\mathrm{BR})//64500\;\\ & \;\;\left(\mathrm{molecular}\;\mathrm{weight}\;\mathrm{of}\;\mathrm{Hb}\right)\}\times 4\times 0.2\;\mathrm{dL}/\mathrm{kg}\times 3\;\mathrm{kg}\\ & \;=0.327\;g\;(327\;\mathrm{mg}) \end{array}$$

This load can be adjusted by the time of cord clamping at delivery. Early cord clamping has been reported to decrease the effectiveness of phototherapy in a comparative study of Japanese term infants (80), but other studies have reported different results (81–83). The American College of Obstetricians and Gynecologists has suggested that many newborns—not only preterm infants but also term infants—would benefit greatly from a cord clamping delay of 30–60 s or more (84). However, this recommendation requires consideration of the impact of race on neonatal jaundice. Peak serum bilirubin levels of physiological jaundice are more than twice as high in Asians as in Caucasians, and many Asian newborns develop neonatal hyperbilirubinemia (85–87). If the bilirubin levels requiring treatment are the same, the number of patients treated for the same bilirubin load will be higher in the Asian population. This is also why the hour-specific nomogram of transcutaneous bilirubin concentration, which is used to identify treatment reference subjects, should also be generated for each race (88). In other words, applying the hour-specific nomogram of the transcutaneous bilirubin concentration created for Caucasians to Asians would increase the number of neonates eligible for blood sampling.

Erythrocyte lifespan is believed to be shortened in neonates due to differences between the membranes of neonatal and adult erythrocytes. The relationship between fetal hemoglobin and erythrocyte lifespan has been examined with respect to subunit interface strength. Erythrocytes with a weaker interface strength have a shorter lifespan. The dimer interface strength of fetal hemoglobin is weaker than that of adult hemoglobin, representing maturation from weaker to stronger monomer-monomer subunit contacts (89). Neonatal erythrocyte membranes have been reported to exhibit mechanical fragility (90). We compared ROS production in response to oxidative stress between cord blood and maternal erythrocytes *in vitro*. The production of ROS was significantly higher in cord blood erythrocytes than in maternal erythrocytes (91). The low vitamin E content (92, 93) and high lipid composition with high levels of active hydrogen in neonatal erythrocyte membranes is believed to be the cause. However, the reason for the significant positive correlation of ROS production between the mother and neonate is unknown. Regarding racial differences in neonatal bilirubin production, one study found significantly higher levels of serum carboxyhemoglobin in Japanese neonates than in Caucasian neonates (94). Thus, bilirubin is thought to exert some physiological effects due to its increased production in the neonatal period, causing neonatal jaundice.

**Figure 5:**
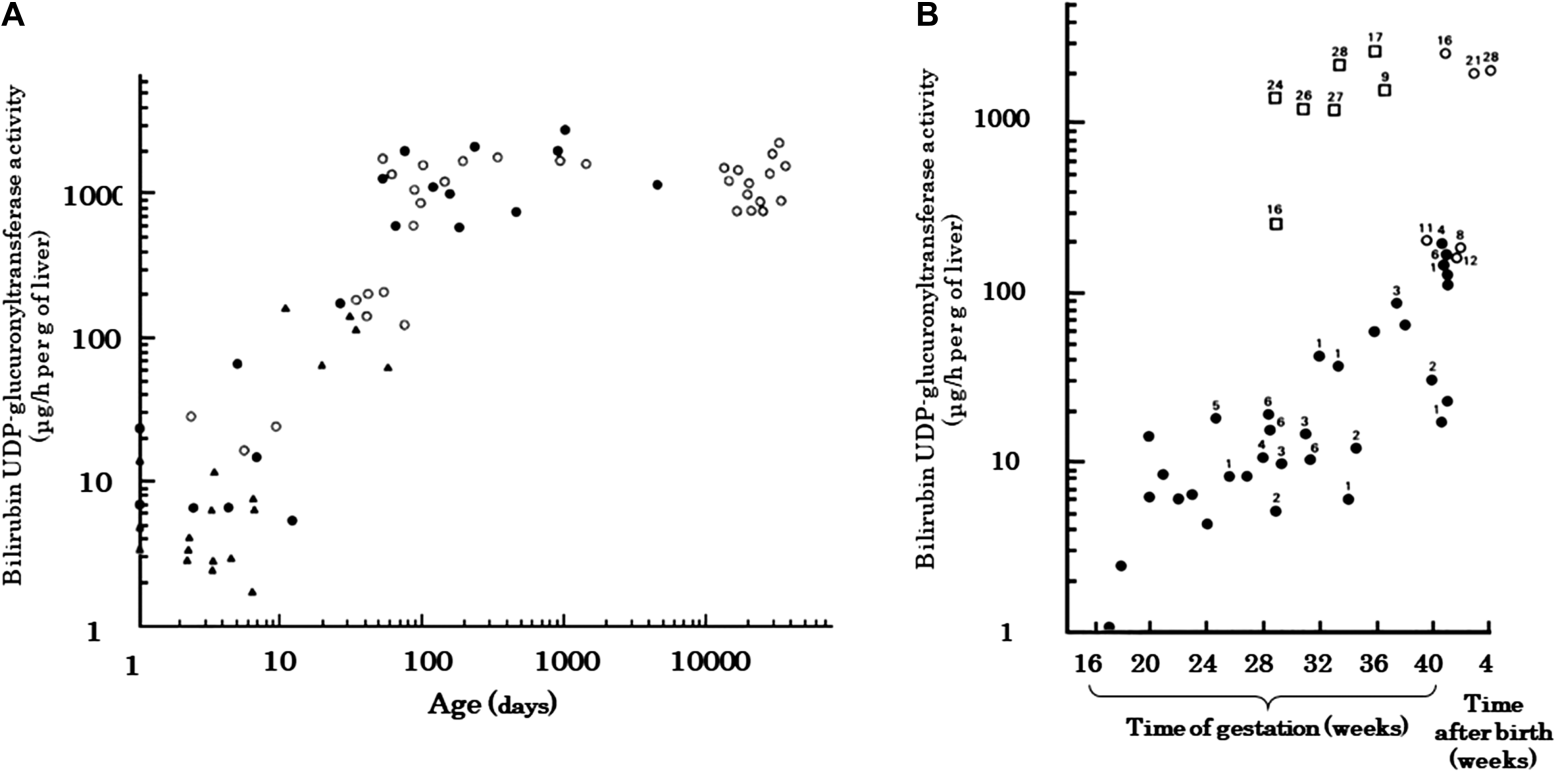
Developmental change in human hepatic bilirubin UDP-glucuronosyltransferase activity [adapted from onishi et al. (95) and Kawade and Onishi (68)]. (**A**) Measurements from newborn to adult. Both the ordinate and the abscissa are plotted on logarithmic scales. ●, autopsied cases; ○, upper abdominal laparotomy; ▴, preterm neonates. (**B**) Relationship between enzyme activity and survival. Preterm delivery, irrespective of gestational age, evokes an early increase in transferase activities, equal in rate to the normal postnatal increase. The number shown beside the symbols represent the age (days) at which death occurred. Transferase activities are also shown for preterm (□) and term (○) infants who lived more than 8 days after birth. ●, Transferase activities for fetuses and preterm and term infants who died within 7 days of birth.

An important aspect of the metabolic pathway for bilirubin excretion is the bilirubin conjugating capacity of the liver. The capacity in the liver of term neonates is about 1% of that of adults, with a rapid increase in activity at birth that lasts until 90 postnatal days, after which it plateaus until adulthood (Figure 5A) (95). It has also been demonstrated that the activity increases after birth in preterm infants (Figure 5B) (68). Many studies have reported the effects of genetic polymorphisms in *UGT1A1* on neonatal jaundice, including breast milk jaundice and preterm jaundice, with *UGT1A1 TATA*-box polymorphism (96), UGT1A1*6 (97–100), and UGT1A1*28 (101) as the reported jaundice enhancers. As an excretion route other than conjugation, photoequilibrium between BR and *4Z,15E-*bilirubin IXα is established in bright environments, and *4Z,15E*-bilirubin IXα is excreted into the bile without conjugation. However, *4E,15Z*-cyclobilirubin IXα is produced at low amounts, depending on the amount of ambient light energy (26, 39, 102). Conjugated bilirubin and photoisomers of bilirubin excreted in the bile can be reconverted to BR in the gastrointestinal tract and are reabsorbed in the intestines, where they enter the enterohepatic circulation. Conjugated bilirubin is metabolized back to BR by the underdeveloped intestinal microbiome and high β-glucuronidase activity in the intestinal tract and breast milk (103). The free *4Z,15E*-bilirubin IXα is easily reconverted to the BR in a process that requires energy, such as body temperature (37). Thus, the neonatal period is designed to store BR *in vivo*.

The enterohepatic circulation of this bilirubin is believed to play an important role in breast milk jaundice, which is considered physiological. Reported causes of breast milk jaundice include inhibitors of hepatic *UGT1A1* in breast milk (pregnane-3α, 20β-diol, non-esterified fatty acid), increased enterohepatic circulation (β-glucuronidase, epidermal growth factor), genetic polymorphisms (*UGT1A1* mutations such as UGT1A1*6 and UGT1A1*28), dehydration, starvation, IL-1β, and α-fetoprotein (97–101, 104–106). Of these, the elevation of the enterohepatic circulation is reported to be due to the presence of β-glucuronidase (103) and epidermal growth factor (107) in breast milk, and the influence of inhibitors of intestinal *UGT1A1* activity in breast milk has also been identified recently. In neonatal *hUGTI* mice, *UGT1A1* metabolizes bilirubin in the gastrointestinal tract to prevent the accumulation of bilirubin. Intestinal *UGT1A1* is under control by IKK/NF-кB signaling. Breast milk can inhibit the NF-кB mediated transcription of *UGT1A1* (108).

When considering the antioxidant role of bilirubin in neonates, it is necessary to consider the extent to which bilirubin acts relative to many low-molecular-weight antioxidants. In terms of antioxidant concentrations, neonates have higher levels of bilirubin, uric acid, and vitamin C, but lower levels of vitamin E, carotenoids, and vitamin A compared with adults, and bilirubin is often present at the highest concentration among these antioxidants. However, albumin, haptoglobin, and hemopexin, which are proteins with antioxidant properties, are at low levels, while transferrin is high in response to Fe^2+^, which tends to increase in the neonatal period (109). Serum bilirubin concentrations have been studied in relation to the reduction in antioxidant capacity and oxidants (Table 1) and in relation to diseases caused by ROS damage. The relationship between serum bilirubin and total antioxidant status appears to be affected by low concentrations of bilirubin, and the status is remarkably robust at high concentrations of bilirubin. However, conflicting results have been obtained for total oxidant status, which is expected to decrease with elevation of bilirubin due to its antioxidant effect, which increases with bilirubin concentration (110, 111). Neonatal diseases that develop due to ROS damage, including retinopathy of prematurity, intraventricular hemorrhage, necrotizing enterocolitis, chronic lung disease (bronchopulmonary dysplasia), and sepsis or severe fungal infection, were evaluated *via* a comparison of bilirubin levels with those of controls. The results did not provide sufficient evidence that bilirubin prevents the development of these diseases through its antioxidant effect (112, 113). When considering the antioxidant effects of bilirubin, it is necessary to examine the relationship between the decrease in bilirubin and the increase in BR oxidation products. In basic *in vitro* studies of phototherapy light sources for neonatal hyperbilirubinemia, the reduction in BR levels with light irradiation was thought to be mainly due to photochemical changes (Figure 2) and also due to a small amount of cyclobilirubin polymerization and BR oxidation products. Under conditions with minimal cyclobilirubin polymerization, the amount of BR oxidation products is [amount of BR reduced−amount of total BR photoisomer]. Under these conditions, a comparison of the phototherapy light source with blue-white and green fluorescent light demonstrated that there are fewer BR oxidation products with green light under the addition of flavin mononucleotide (58). In addition, methylxanthine derivatives exhibited enhanced production of BR oxidation products under blue-white fluorescent light irradiation in the presence of flavin mononucleotide (114). The Umu test, which measures DNA mutagenicity, was also significantly higher under blue-white fluorescent light compared with green fluorescent light (115).

**Table 1:** Biomarkers of oxidative stress and antioxidation.

**1. Antioxidant capacity** (1) Water soluble: Plasma total antioxidant capacity, total SH, GSH, VC (2) Fat soluble: CoQ_10_ oxidation rate, lutein, zeaxanthin, β-cryptoxanthin, lycopene, alpha-carotene, β-carotene, vitamin A, vitamin E fraction, α-tocopherol/cholesterol
**2. Oxidation potential**
Serum total oxidant status (1) DNA: 8-OHdG, apurinic/apyrimidinic site, nitroguanosine, thymidine glycol (2) RNA: microRNA (3) Lipid: MDA, LPO, oxidized LPO, HEL, isoprostane, total hydroperoxide, neuroprostanes, isofurans, neurofurans (4) Sugar: CML, 3-DG, albumin glycoside, pentosidine (5) Protein: AOPP, carbonylated protein
**3. NO**
Nitrotyrosine
**4. Antioxidation and inflammation-related enzymes**
SOD, CAT, GSH peroxidase, MPO
**5. Redox Anti-Aging**
PON-1, TRX, PRX

GSH, glutathione; VC, vitamin C; CoQ_10_, coenzyme Q10; 8-OHdG, 8-hydroxy-2’-deoxyguanosine; MDA, malondialdehyde; LPO, lipid peroxide; HEL, Nε-(Hexanoyl) lysine; CML, carboxymethyllysine; 3-DG, 3-deoxyglucosone; AOPP, advanced oxidation protein products; SOD, superoxide dismutase; CAT, catalase; MPO, myeloperoxidase; PON-1, paraoxonase; TRX, thioredoxin; PRX, peroxiredoxin.

Daylight irradiation is reported to cause DNA damage *in vitro*, which was attributed not to bilirubin but to ROS generated by endogenous substances (116, 117). *In vivo* effects include those seen in a famous animal study where Gunn rats were treated with riboflavin-5-phosphate and exposed to blue light, which caused adverse reactions such as blistering and bleeding (56). These results are also thought to be due to ROS produced by the photosensitizing effect of riboflavin irradiated with light at sensitive wavelengths.

Regarding breast milk jaundice, vitamin A and unsaturated fatty acids, which were first noted for their antioxidant effects on bilirubin, may act as stabilizers in the intestinal tract (1–5). In our previous study, there was a significant positive correlation between serum BR and urinary propentdyopent reactive substances (31). Breast milk jaundice may also have some physiological effect due to the antioxidant effects of bilirubin. We hope that further progress in the identification of BR oxidation products will clarify the role of bilirubin in diseases caused by ROS toxicity in the neonatal period.

## 7. Conclusion

Bilirubin metabolism in the fetus and neonate is centered on BR. The physiological significance of neonatal jaundice, including breast milk jaundice, which occurs only in humans and rhesus monkeys, needs to be studied in terms of antioxidant effects. Phototherapy for neonatal hyperbilirubinemia requires the development of safe and effective light sources that do not increase BR oxidation products, and it is also necessary to determine a safe threshold for the bilirubin level that does not exert bilirubin toxicity but still has antioxidant effects.

## Author contributions

SI contributed to the conception and design of this review and drafted the manuscript. HO, YK, and TI prepared the figures and table. KK, SN, and TK performed English editing and literature review. TK critically reviewed the manuscript. All authors contributed to the article and approved the submitted version.
